# The Diploma-Ed Danger

**Published:** 1892-10

**Authors:** 


### THE DIPLOMA-ED DANGER.

Much has been said and written about the danger to the public, of permitting anybody and everybody who calls himself "Doctor," to practice medicine. The cry for a bill to "regulate the practice" has been long and loud;—successful in some, unavailing in other States, — and in Texas methinks I hear the faint echo of the last cry dying away in the distance like the wail of despair from a lost spirit; but do we recognize the fact, that amongst those armed with *the one* desideratum—*the* qualification *par excellence*, the open-sesame to all medical societies,—the *Diploma*, the *license* to deal with life and death, and the warrant to kill,—there are ignorant men, no more competent to practice medicine than the branded quack, and that they get in their deadly work daily, and with entire impunity, and go forever unpunished, suffering not even a pang of conscience, because they do not *know* they are ignorant? and others who, not even knowing the proper doses of the poisonous drugs, kill people, and ascribe the death to the disease, or to causes other than their guilty ignorance? It is too true; an overdose of morphine will forever hush the cry of the sick baby; the mother's heart may break—the vacant chair, the broken doll, serving as a perpetual reminder of the loved and lost, and the blame (for somebody or something must be blamed) is calmly, and with religious (?) unction, laid to the "Lord;"—"The Lord giveth and the Lord taketh away," the hypocritic, but diploma-ed doctor will say; or, "Too bad! If I had only been called a little earlier;" or, "The vital powers, madam, were overtaxed, and the disease had made such headway that science was powerless to avert it." Oh science, as Madame DeStael said of liberty, how many sins are committed in thy name!

The foregoing reflections have been suggested by reading in the Kansas City *Med. Index* the report of a case where a *baby seven months old* had received a scald on the arm and cheek, for which "Dr. C." (who reports the case!) prescribed "Carbolic acid, camphor gum, aa 1 drachm; to castor oil, ℥j;" and gave *⅛ gr. of*

*morphine*, and left another ⅛ grain, "to be given in two or three hours, if necessary." The reporter coolly says: "*The child dropped to sleep and died in about three hours without awakening*," yet as coolly attributes the death to the carbolic acid, and discusses the probability of its having been absorbed!

We do not know which to admire most, the Doctor's childlike, ignorant simplicity, or his assumption that every body else is as big a fool as he; for it is evident that *he* never suspected the overdose of morphine had done its work, or he would not have given it away. It is a remarkable report.

